# Glycyrrhizic Acid Alleviates Atherosclerosis in *ApoE*^−/−^ Mice via Microbial Indole-3-Lactic Acid-Mediated AhR-p65 Interaction in the Endothelium

**DOI:** 10.3390/ijms27156694

**Published:** 2026-07-27

**Authors:** Haoran Shen, Shuai Huang, Zhiyu Wang, Sitong Zhou, Lulu Huang, Hongjuan Zhang, Yanxing Han, Jiandong Jiang, Huihui Guo

**Affiliations:** 1 State Key Laboratory of Bioactive Substance and Function of Natural Medicines, Institute of Materia Medica, Chinese Academy of Medical Sciences & Peking Union Medical College, Beijing 100050, China; shenhaoran@imm.ac.cn (H.S.); wangzhiyu@imm.ac.cn (Z.W.); zhousitong@imm.ac.cn (S.Z.); zhjuan@imm.ac.cn (H.Z.); hanyanxing@imm.ac.cn (Y.H.); 2 Biomedical Innovation Center, Beijing Shijitan Hospital, Capital Medical University, Beijing 100038, China; huangshuai@bjsjth.cn; 3 Hubrecht Institute, Royal Netherlands Academy of Arts and Sciences (KNAW) and University Medical Center Utrecht, 3584 Utrecht, The Netherlands; l.huang@hunrecht.eu; 4 Institute of Medicinal Biotechnology, Chinese Academy of Medical Sciences & Peking Union Medical College, Beijing 100050, China

**Keywords:** glycyrrhizic acid, atherosclerosis, microbiota–vascular axis, indole-3-lactic acid, endothelial inflammation

## Abstract

Glycyrrhizic acid (GL), a natural triterpenoid glycoside extracted from the “medicine food homology” herb *Glycyrrhiza glabra* L., exhibits potent anti-atherosclerotic effects; yet its underlying mechanisms remain unclear due to its poor oral bioavailability. The gut microbiota plays a pivotal role in the development of atherosclerosis (AS). In this study, the microbiota-dependent anti-AS effects of GL were evaluated in high-fat diet (HFD)-fed *ApoE*^−/−^ mice using antibiotic depletion and fecal microbiota transplantation (FMT). Integrated metagenomic and metabolomic analyses were performed to identify the key bioactive microbial metabolite. Further in vivo and in vitro experiments, including co-immunoprecipitation and dual-luciferase reporter assays, were utilized to elucidate the underlying molecular mechanisms. It was demonstrated that oral administration of GL alleviated AS in a microbiota-dependent manner by reversing gut dysbiosis, improving intestinal barrier function, and reducing pro-inflammatory lipopolysaccharide (LPS) levels. GL shifted intestinal tryptophan metabolism toward bacterial-derived indole-3-lactic acid (ILA) production, suppressing LPS-induced vascular endothelial adhesion dysfunction by activating the aryl hydrocarbon receptor (AhR). Mechanistically, ILA-activated AhR interacted with the NF-κB subunit p65 in the cytoplasm, effectively preventing the nuclear translocation of p65 and suppressing the promoter activities of adhesion molecules (*VCAM1* and *ICAM1*), resulting in the amelioration of HFD-induced AS. These findings elucidate the microbiota-dependent mechanism of orally administered GL against AS, and highlight the therapeutic potential of targeting the ILA-AhR-p65 axis in the vascular endothelium as a strategy for AS.

## 1. Introduction

Cardiovascular disease (CVD) is the leading cause of morbidity and mortality worldwide [1]. Atherosclerosis (AS), a lipid-driven inflammatory disease of the arterial wall initiated by endothelial activation and sustained by persistent immune infiltration, is the primary pathological basis of CVD [2,3]. Currently, based on the patient’s condition, AS treatment predominantly involves surgery and medical therapy. Although surgical treatment effectively reduces adverse outcomes and mortality in selected patients with advanced vascular disease, it exerts high demands on the patient’s vital signs and may cause bleeding complications or postoperative restenosis [4,5], suggesting that medical treatments are more convenient and extensive therapies. As the first-choice drugs for lowering low-density lipoprotein cholesterol (LDL-C), statins effectively reduce AS-induced major adverse cardiovascular events individually or in combination with proprotein convertase subtilisin/kexin type 9 inhibitors (evolocumab), cholesterol absorption inhibitors (ezetimibe), platelet adhesion inhibitors (aspirin) and/or antioxidants (probucol). These current medicines are regarded as the cornerstone of CVD prevention and treatment. However, the therapeutic benefits may be compromised owing to poor drug tolerance in a sizeable number of AS patients [6]. For example, statin-induced adverse effects such as myalgia, hepatotoxicity, new-onset diabetes, and probucol-induced potential cardiotoxicity restrict their clinical application for some intolerant patients [7,8]; aspirin is also not suitable for elderly over 70 years old due to the high risk of bleeding [9]. More importantly, even with optimal lipid reduction, a substantial residual inflammatory risk persists in many patients, driving continued plaque progression [10,11]. Thus, novel therapeutic strategies that can overcome these limitations are urgently needed.

Over recent decades, accumulating evidence has shown that the gut microbiota are associated with the progress of various AS-related conditions, including hypertension, diabetes, hyperlipidemia, obesity, and arterial thrombosis [12,13,14]. The link between the gut microbiota and AS is primarily driven by two factors: intestinal barrier integrity and microbial metabolites. First, the disruption of the intestinal epithelial barrier permits the systemic translocation of bacteria-derived lipopolysaccharide (LPS), serving as a persistent pro-inflammatory stimulus for the vascular endothelium. Second, some microbial metabolites enter the systemic circulation to function as signaling molecules [15]. For example, the increased abundance of *Klebsiella pneumoniae* facilitates the conversion of dietary choline to trimethylamine (TMA), which can subsequently be oxidized to pro-atherosclerotic trimethylamine N-oxide (TMAO) in the liver, leading to abnormal cholesterol metabolism and insulin resistance, then inducing platelet aggregation and thrombosis [16,17]. Conversely, beneficial metabolites exert profound atheroprotective effects. Microbial-derived short-chain fatty acids (SCFAs) effectively increase hepatic and vascular endothelial ATP binding cassette transporter A1 (ABCA1) expression, which may accelerate reverse cholesterol transport and thus prevent the development of AS [18]. Nowadays, bacterial-produced indole derivatives have emerged as key regulators in maintaining vascular endothelial homeostasis. For instance, indole-3-carboxaldehyde (IAld) activates the endothelial Nrf2/HO-1 pathway via the aryl hydrocarbon receptor (AhR) signaling, leading to a reduction in pro-atherosclerotic cytokines and oxidative stress [19]; indole-3-acetic acid (IAA) attenuates vascular inflammation by modulating the TLR4/MyD88 signaling [20].

Endothelial dysfunction is widely recognized as an initial event in the pathogenesis of AS [21]. The impaired vascular endothelium upregulates critical adhesion molecules, prominently vascular cell adhesion molecule-1 (VCAM-1) and intercellular adhesion molecule-1 (ICAM-1), which act as crucial mediators for the recruitment and intimal infiltration of circulating monocytes and macrophages [22]. Canonical TLR4/NF-κB signaling pathway affects the expression of ICAM-1 and VCAM-1. Triggered by LPS leaked from the compromised intestinal barrier, the activation of TLR4 and subsequent proteasome-mediated degradation of IκB liberates the NF-κB p65 subunit, driving its nuclear translocation to activate the transcription of these adhesion molecules [23]. AhR signaling pathway, activated by microbial-derived indoles, was reported to effectively suppress this progress. As a ligand-dependent transcription factor, AhR translocates into the nucleus and forms a complex with the AhR nuclear translocator (ARNT) to directly regulate the expression of target genes [24]. Emerging evidence has highlighted a non-canonical AhR signal transduction. The AhR–agonist complex regulates the activity of other transcription factors [25]. For example, microbial-derived indole-3-propionic acid (IPA) effectively inhibits NF-κB activity by reducing the ubiquitination of IκB via AhR-β-TrCP interaction. However, whether gut microbiota-derived indoles can mitigate vascular adhesion and alleviate AS through this non-canonical transrepression mechanism within the vascular endothelium remains largely unexplored.

Glycyrrhizic acid (GL), a natural triterpenoid glycoside extracted from the “medicine food homology” herb *Glycyrrhiza glabra* L., is widely used as a natural sweetener, and exhibits good safety for long-term administration [26]. GL is clinically applied for hepatoprotection in China and other countries. Modern pharmacological studies have revealed the potential of GL for the alleviation of AS in both oral and injected formulations by inhibiting the inflammatory response, oxidative stress, and by improving dyslipidemia and vascular endothelial function [27,28,29,30], suggesting that GL can be a complementary option for those AS patients who are intolerant to the current CVD drugs. However, due to its poor absorption and bioavailability, the oral systemic concentration of GL (<1 μg/mL) cannot achieve that obtained for many of the known pharmacological effects in vitro (2–40 μg/mL) and intravenous injection (20–50 μg/mL), thus suggesting that the mechanism underlying the effect of oral GL during the treatment of AS differed from the injection and the direct regulation of related signaling pathways [28,29,30,31]. Considering the long treatment period of AS and medication compliance, oral GL is much more suitable than an injected preparation. Thus, investigating the underlying mechanism of oral GL against AS is crucial for the clinical application of GL.

A large amount of the GL that is not absorbed subsequently interacts with the gut microbiota; this is important because microbial metabolism may critically mediate therapeutic outcomes. In this study, we aimed to identify the mechanism of GL against AS through the gut microbiota and bacterial metabolites using an integrated microbiome-metabolome analysis in *ApoE*^−/−^ mice fed a high fatty diet (HFD). We revealed that GL effectively improved the intestinal barrier, reduced circulating LPS levels and remodeled the gut microbiota to promote microbial tryptophan-indole metabolism, leading to a profound enrichment of indole-3-lactic acid (ILA). Mechanistically, ILA-activated AhR physically interacted with the NF-κB subunit p65 in the cytoplasm, effectively preventing its nuclear translocation and suppressing the promoter activities of adhesion molecules (*VCAM1* and *ICAM1*), leading to the alleviation of AS. Our findings provide the first evidence that oral GL ameliorates AS by modulating the microbiota–vascular axis specifically through the ILA-AhR-p65 molecular crosstalk.

## 2. Results

### 2.1. Oral GL Effectively Ameliorated AS in HFD-Fed ApoE^−/−^ Mice

**Figure 1:**
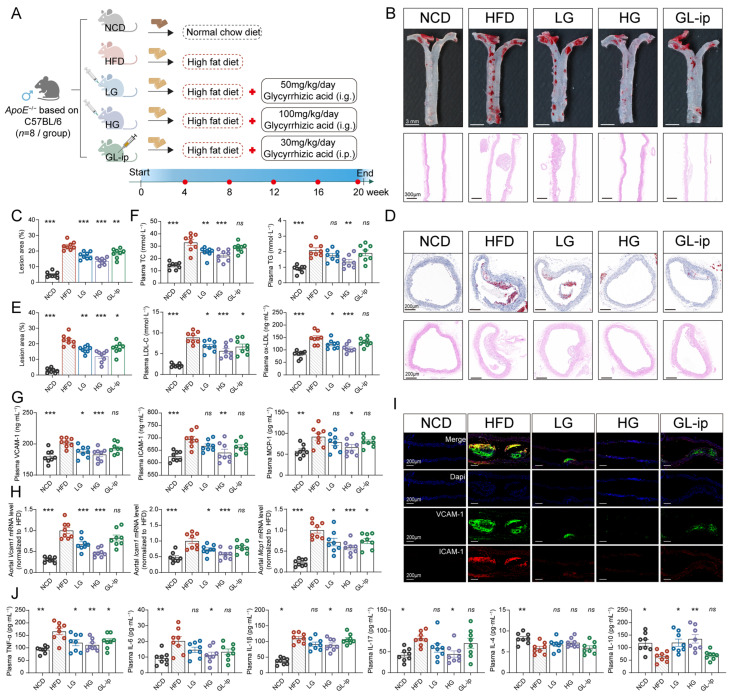
Oral GL effectively ameliorated AS in HFD-fed *ApoE*^−/−^ mice. (**A**) Schematic diagram of experimental design for administering GL; (**B**) Representative images of oil red O-stained (top, scale bars: 3 mm) and hematoxylin and eosin (H&E)-stained (bottom, scale bars: 300 μm) longitudinal aorta; (**C**) Quantification (%) of oil red O-stained longitudinal aorta; (**D**) Representative images of oil red O-stained (top, scale bars: 200 μm) and H&E-stained (bottom, scale bars: 200 μm) transverse aortic roots; (**E**) Quantification (%) of oil red O-stained aortic roots; (**F**) The levels of TC, TG, LDL-C and ox-LDL in plasma. (**G**) The levels of ICAM-1, VCAM-1, and MCP-1 in plasma; (**H**) Relative mRNA level of arterial *VCAM-1*, *ICAM-1*, and *MCP-1*; (**I**) Representative images of immunofluorescence staining for VCAM-1 (green) and ICAM-1 (red) with DAPI counterstaining for nuclei (blue) in the aorta of *ApoE*^−/−^ mice (scale bars: 200 μm); (**J**) The levels of plasma TNF-α, IL-6, IL-1β, IL-17, IL-4 and IL-10 as determined by Luminex. *n* = 8. Data are presented as means ± SEM, * *p* < 0.05, ** *p* < 0.01, *** *p* < 0.001, ns: no significant difference, using ANOVA.

In this study, HFD-fed *ApoE*^−/−^ mice were used to evaluate the efficacy of GL on AS (Figure 1A). Considering the potential adverse effects of GL on blood pressure, metal ion, aldosterone, and renin levels, we first evaluated its safety profile. After treatment for 20 weeks, we did not observe obvious toxicity on kidney (Figure S1A,B). Moreover, none of plasma K^+^, Na^+^, renin or aldosterone levels were significantly altered after GL treatment, suggesting that GL had no effect on blood pressure (Figure S1C,D). These results revealed the good safety of GL in HFD-fed *ApoE*^−/−^ mice. We believe that the decreased activity of bacterial β-glucuronidase, an enzyme that metabolizing GL into toxic 18β-glycyrrhetinic acid, in the high-fat diet group might be an important factor for maintaining its long-term safety (Figure S1E).

Oil red O and hematoxylin and eosin (H&E) staining of the longitudinal aorta showed that oral GL effectively attenuated atherosclerotic plaques and lipid deposition (32% reduction by LG, 46% reduction by HG) (Figure 1B,C). Consistent results were also observed for the staining of transverse aortic roots (Figure 1D,E). We also evaluated the plasma lipid parameters of *ApoE*^−/−^ mice and found that oral GL significantly reduced the levels of TC, LDL-C and ox-LDL (Figure 1F). AS is often accompanied by dysfunction of the arterial vascular endothelium, such as the upregulated expression of MCP-1, ICAM-1 and VCAM-1, thus resulting in the adhesion of monocytes to the endothelium and inflammatory infiltration [32,33]. In the present study, we found that oral GL significantly alleviated the HFD-induced elevation in ICAM-1, VCAM-1, and MCP-1 levels both in the plasma and aorta (Figure 1G–I). Analysis of inflammatory infiltration showed that oral GL effectively inhibited the release of pro-inflammatory factors (TNF-α, IL-6, IL-1β and IL-17) and increased the production of anti-inflammatory cytokines (IL-4 and IL-10), especially with regard to the level of IL-10 (Figure 1J). These results demonstrated that oral GL effectively ameliorated AS and related risk factors.

Moreover, we comparatively evaluated the efficacy of intraperitoneal GL against AS, and found that intraperitoneal GL also inhibited the progression of HFD-induced AS, although it appeared to show a relatively weaker effect than oral GL (Figure 1). Considering the low absorption of oral GL, we further determined plasma drug–time curves for oral and intraperitoneal GL, and observed that the *C_max_* for oral administration (<1 μM) was much lower than that for intraperitoneal injection (≈20 μM) (Figure S2A). Subsequently, in vitro results showed that low concentrations of GL (0.5 μM, 1 μM) barely affected the expression of adhesion factors (ICAM-1 and VCAM-1). Even at a high concentration (20 μM), GL only slightly reduced these adhesion markers, and the effect did not reach statistical significance (Figure S2B). These data indicate that the anti-atherosclerotic effect of oral GL is unlikely mediated by the direct inhibition of endothelial adhesion molecules, further supporting a microbiota-dependent mechanism distinct from that of intraperitoneal GL.

### 2.2. The Gut Microbiota Participated in the Anti-AS Effects of GL

**Figure 2:**
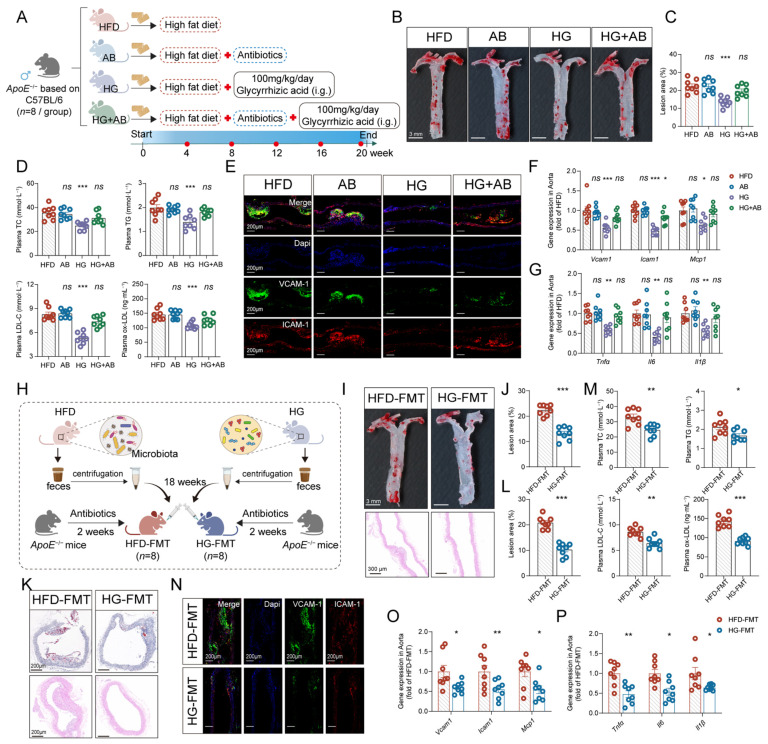
The gut microbiota participated in the anti-AS effects of GL. (**A**) Schematic diagram of experimental design for AB strategy; (**B**,**C**) Representative images and quantification (%) of oil red O-stained aorta. (scale bars: 3 mm); (**D**) The levels of TC, TG, LDL-C, and ox-LDL in plasma; (**E**) Representative images of immunofluorescence staining for VCAM-1 (green) and ICAM-1 (red) with DAPI counterstaining for nuclei (blue) in the aorta (scale bars: 200 μm); (**F**) Relative mRNA level of arterial *Vcam1*, *Icam1*, and *Mcp1*. (**G**) Relative mRNA level of arterial *Tnfα*, *Il6* and *Il1β*; (**H**) Schematic diagram of experimental design for FMT strategy; (**I**) Representative images of oil red O-stained (top, scale bars: 3 mm) and H&E-stained (bottom, scale bars: 300 μm) longitudinal aorta; (**J**) Quantification (%) of oil red O-stained longitudinal aorta; (**K**) Representative images of oil red O-stained (top, scale bars: 200 μm) and H&E-stained (bottom, scale bars: 200 μm) transverse aortic roots; (**L**) Quantification (%) of oil red O-stained aortic roots; (**M**) The levels of TC, TG, LDL-C and ox-LDL in plasma; (**N**) Representative images of immunofluorescence staining for VCAM-1 (green) and ICAM-1 (red) with DAPI counterstaining for nuclei (blue) in the aorta (scale bars: 200 μm); (**O**) Relative mRNA level of arterial *Vcam1*, *Icam1*, and *Mcp1*; (**P**) Relative mRNA level of arterial *Tnfα*, *Il6* and *Il1β*. *n* = 8. Data are presented as means ± SEM, * *p* < 0.05, ** *p* < 0.01, *** *p* < 0.001, ns: no significant difference. For two groups, the F test was used to test homoscedasticity, and significance was calculated by unpaired Two-tailed Student’s *t*-test.

The low oral bioavailability of GL results in substantial gastrointestinal retention, enabling direct interaction of GL with gut microbiota. To investigate potential microbiota-dependent mechanisms underlying the anti-AS effects of GL, we used antibiotic cocktail (AB) therapy to evaluate the contribution of the gut microbiota to the amelioration of AS and related metabolic disorders by GL (Figure 2A). The use of antibiotics neither affected the conditions of *ApoE*^−/−^ mice nor altered the AS-related indicators. However, the removal of gut microbiota markedly weakened the efficacy of GL in alleviating AS including aortic plaque area, lipid levels, and aortic inflammatory-related genes (Figure 2B–G), suggesting that the gut microbiota may participate in the anti-AS effects of GL.

To further demonstrate the role of the gut microbiota in the anti-AS effect of GL, we next performed FMT from HFD or HG-treated donor *ApoE*^−/−^ mice to AB-pretreated HFD recipient *ApoE*^−/−^ mice (HFD-FMT, HG-FMT) by oral gavage (Figure 2H). Analysis showed that HG-FMT mice exhibited significant attenuation of atherosclerotic pathology when compared to the HFD-FMT group, with a 44% reduction in the total area of atherosclerotic plaques and a 51% reduction in aortic root lesions (Figure 2I–L). The levels of plasma lipid (Figure 2M), endothelial ICAM-1/VCAM-1 (Figure 2N,O), and aortic inflammatory cytokines (Figure 2P) were also ameliorated in the HG-FMT group, revealing that HG-FMT recapitulated the therapeutic benefits observed in GL-treated donor mice. The collective evidence for both the FMT and AB strategy highlight the critical role of the gut microbiota in the ability of GL to ameliorate AS.

### 2.3. GL Reshaped the Gut Microbiota and Enriched Tryptophan-Metabolizing Bacteria

**Figure 3:**
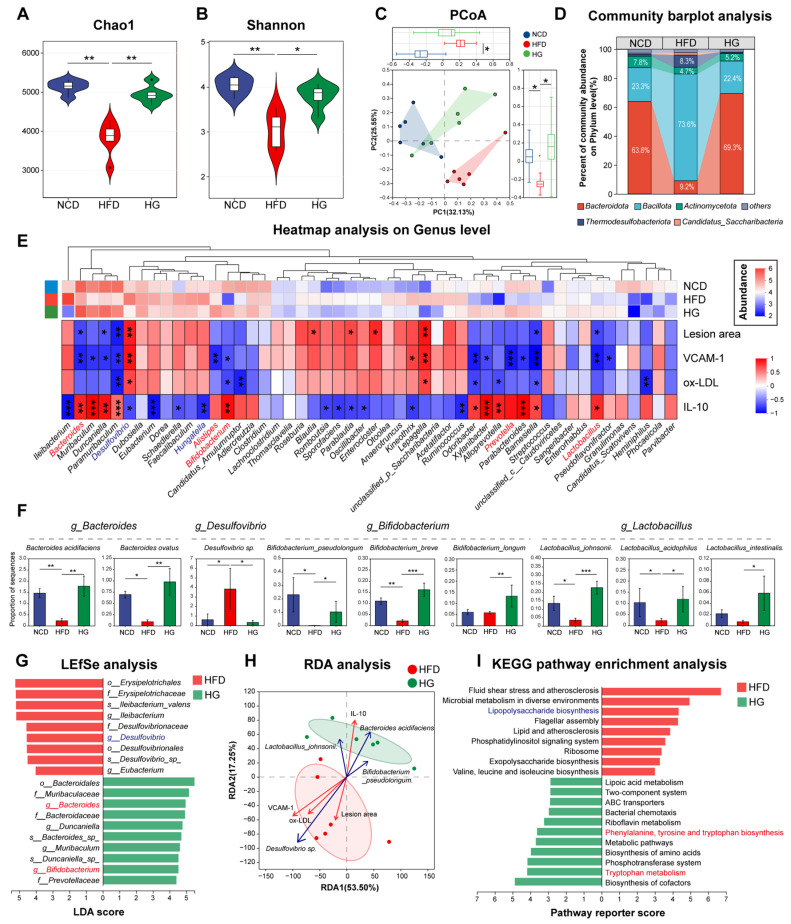
GL reshaped the gut microbiota and enriched tryptophan-metabolizing bacteria. (**A**,**B**) Multisample Chao1 index (**A**) and Shannon index (**B**) of each group; (**C**) Microbiota community analysis based on principal coordinate analysis (PCoA); (**D**) Community abundance profiling of the gut microbiota at the phylum level; (**E**) Heatmap of the top 50 genera among NCD, HFD and HG group with Kruskal–Wallis H test (*p* < 0.05); and the Pearson correlation heatmap analysis between genus and AS-related indices with *p* values < 0.05 were considered statistically significant. Red represented a positive correlation (correlation *R* value > 0), and blue indicated a negative correlation (correlation *R* value < 0); (**F**) The quantitation of the abundance of several selected bacteria; (**G**) Linear discriminant analysis effect size analysis (LDA) scores for identifying the key enriched bacteria at different taxonomic levels (from order to species); (**H**) Redundancy analysis (RDA) between the abundance of bacterial species (ameliorated by GL) and the AS-related indices; (**I**) KEGG bubble diagram of enrichment analysis (HG vs. HFD). *n* = 6. Data are presented as means ± SEM, * *p* < 0.05, ** *p* < 0.01, *** *p* < 0.001, using ANOVA.

HFD-induced metabolic disorders, including AS, often result in an imbalance of the intestinal microenvironment, including gut microbiota disturbance and intestinal barrier dysfunction. In this study, we performed shotgun metagenomics sequencing analysis to evaluate the effect of GL on the modulation of the gut microbiota. Analysis of α-diversity in the gut microbiota showed that AS mice exhibited a significant reduction in both bacterial richness (Chao1 index) and evenness (Shannon index) compared to NCD mice; however, GL supplementation significantly restored disorders of bacterial α-diversity (Figure 3A,B). Principal coordinate analysis (PCoA) based on Bray–Curtis distances revealed a clear separation among the three groups (Figure 3C). The HFD group clustered apart from the NCD group, whereas the HG group shifted toward the NCD cluster, suggesting that GL treatment reshaped the overall gut microbiota structure.

At the phylum level, HFD induced an increase in the *Bacillota* (*Firmicutes*)/*Bacteroidetes* ratio, a well-recognized feature of gut dysbiosis in metabolic disorders. GL treatment effectively reversed the imbalance (Figure 3D). To further investigate the ability of GL to regulate the gut microbiota, we selected the top 50 most abundant genera and carried out Spearman’s correlation analysis between the abundance of genera and several key parameters of AS, including lesion area, VCAM-1, ox-LDL and IL-10. Analysis indicated that several symbiotic genera exhibited high abundance and significant correlations with AS-related indices. Among them, beneficial taxa such as *Bacteroides*, *Bifidobacterium*, *Lactobacillus*, *Alistipes*, and *Prevotella* were positively associated with IL-10 and negatively correlated with pro-atherogenic markers. Conversely, pro-inflammatory *Desulfovibrio* and *Hungatella* showed the opposite pattern, correlating positively with disease markers and negatively with IL-10 (Figure 3E).

Then, the targeted abundance analysis of several representative species from *Bacteroides*, *Desulfovibrio*, *Bifidobacterium*, and *Lactobacillus* showed that GL treatment significantly increased the abundance of all tested species from the beneficial genera, while markedly reducing the abundance of *Desulfovibrio* sp. (Figure 3F). LEfSe analysis (LDA score > 4.0) further identified these genera as key discriminators between the HFD and HG groups (Figure 3G). Notably, several of these genera, including *Bacteroides*, *Bifidobacterium*, and *Lactobacillus*, are known to metabolize tryptophan into indole derivatives. Redundancy analysis (RDA) further revealed that these tryptophan-metabolizing bacteria exhibited the most significant associations with all AS-related markers (negative for lesion area, VCAM-1, ox-LDL; positive for anti-inflammatory IL-10) (Figure 3H).

KEGG pathway enrichment analysis was performed to assess the functional consequences of GL-induced microbiota remodeling. Compared with the HFD group, the HG group showed significant enrichment of pathways related to tryptophan metabolism, bacterial chemotaxis, and two-component systems (Figure 3I). In contrast, pathways involved in LPS biosynthesis were prominently enriched in the HFD group. These results suggest that GL not only restores a healthy microbial architecture but also reprograms the metabolic profile of the microbiota, diverting it from pro-inflammatory endotoxin production toward atheroprotective tryptophan metabolism.

### 2.4. GL Improved Intestinal Barrier Integrity and Reduced Systemic LPS Levels

**Figure 4:**
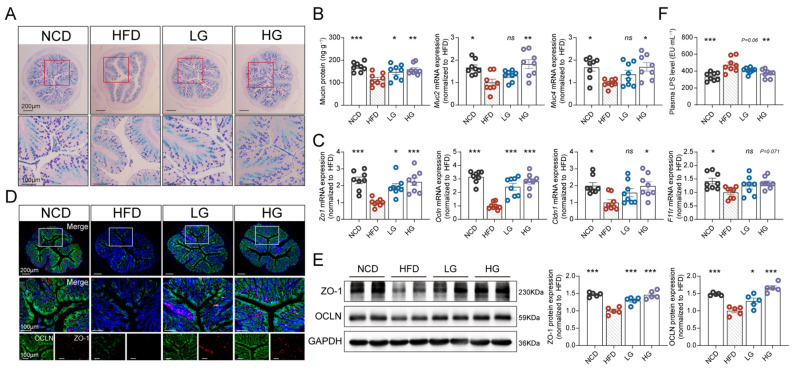
GL improved intestinal barrier integrity and reduced systemic LPS levels. (**A**) Representative images of colonic goblet cells using alcian blue-periodic acid–schiff (AB-PAS) staining (scale bars: 200 μm); (**B**) The mucin protein level (**left**) and relative mRNA level of mucin protein ((**right**), *MUC2* and *MUC4*); (**C**) Relative mRNA level of tight junction (TJ) protein in colonic tissue of *ApoE*^−/−^ mice (*n* = 8); (**D**) Representative immunofluorescence images for ZO-1 (red) and OCLN (green) with DAPI counterstaining for nuclei (blue) in colonic tissue of *ApoE*^−/−^ mice (scale bars: 200 μm); (**E**) Representative Western blots and relative quantitative analysis of the TJ proteins in *ApoE*^−/−^ mice (*n* = 5); (**F**) The level of LPS in plasma (*n* = 8). Data are presented as means ± SEM, * *p* < 0.05, ** *p* < 0.01, *** *p* < 0.001, ns: no significant difference, using ANOVA.

Given that gut microbiota dysbiosis is closely linked to disruption of the intestinal barrier, we next measured the function of the mucus barrier and tight junctions (TJs) in colon tissue to evaluate the effect of GL on gut barrier integrity. Analysis of the mucus barrier characterized by colonic mucin content, expression of *MUC* genes and AB-PAS staining showed that GL effectively repaired the HFD-induced colonic epithelial mucus injury (Figure 4A,B). In addition, the expression levels of key TJ genes including *zonula occludens-1* (*Zo1*), *occludin* (*Ocln*), *Claudin-1* (*Cldn1*), and *junctional adhesion molecule A* (*F11r*) in GL-treated mice were significantly increased when compared with HFD mice (Figure 4C), thus aligning with the results derived from IF staining (Figure 4D) and the Western blotting of ZO-1/OCLN proteins (Figure 4E). Moreover, the level of plasma LPS demonstrated the efficacy of GL on the gut barrier (Figure 4F). These findings revealed that GL effectively ameliorated AS-associated dysbiosis of the gut microbiota and intestinal barrier, thereby improving the overall intestinal microenvironment.

### 2.5. GL Redirected Tryptophan Metabolism to Increase Indole Derivatives

**Figure 5:**
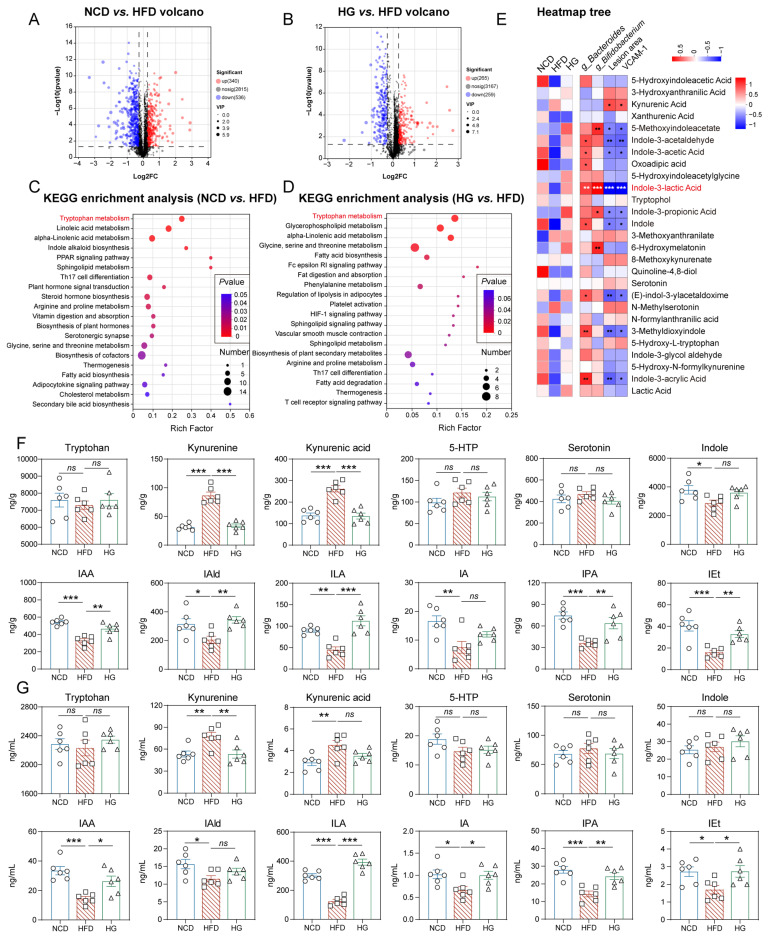
GL redirected tryptophan metabolism to increase indole derivatives. (**A**,**B**) Volcano plot of fecal non-targeted metabolomics of *ApoE*^−/−^ mice from NCD vs. HFD group (**A**), HG vs. HFD group (**B**); (**C**,**D**) KEGG bubble diagram of enrichment analysis in NCD vs. HFD group (**C**) and HG vs. HFD group (**D**); (**E**) Heatmap of the tryptophan metabolites among NCD, HFD and HG group with Kruskal–Wallis H test (*p* < 0.05); and the Pearson correlation heatmap analysis between metabolites and AS-related indices with *p* values < 0.05 were considered statistically significant. Red represented a positive correlation (correlation *R* value > 0), and blue indicated a negative correlation (correlation *R* value < 0); (**F**) Fecal tryptophan-targeted metabolomics analysis in GL-treated *ApoE*^−/−^ mice; (**G**) Plasma tryptophan-targeted metabolomics analysis in GL-treated *ApoE*^−/−^ mice. *n* = 6. Data are presented as means ± SEM, * *p* < 0.05, ** *p* < 0.01, *** *p* < 0.001, ns: no significant difference, using ANOVA.

Bacterial-derived metabolites are regarded as important factors that allow the gut microbiota to alter the progression of diseases. Thus, we conducted untargeted metabolomic profiling of mouse feces to evaluate whether GL significantly affected bacterial metabolism. Principal component analysis (PCA) revealed distinct metabolic clustering among experimental groups in both positive and negative ion modes (Figure S3). Orthogonal partial least squares-discriminant analysis (OPLS-DA) further confirmed robust group separation, with validation parameters (R^2^Y and Q^2^) > 0.65. Comparative analysis identified 876 differentially abundant metabolites (340 upregulated and 536 downregulated) between the NCD and HFD groups (Figure 5A). Metabolic sets were established for these differential metabolites, and KEGG analysis revealed significant enrichment of the tryptophan metabolic pathway (Figure 5B). Similarly, comparison between the HFD and HG groups revealed 524 altered metabolites (265 upregulated and 259 downregulated), with parallel enrichment of tryptophan metabolic pathways (Figure 5C,D). Furthermore, Spearman’s correlation analysis demonstrated that tryptophan-related metabolites, particularly indole derivatives, such as indole-3-lactic acid (ILA), indole-3-propionic acid (IPA), indole-3-acetic acid (IAA) and indole, were negative for atherogenic risk markers (ox-LDL and VCAM-1), but positive for *Bacteroides* and *Bifidobacterium* abundance (Figure 5E). These results suggested that GL may promote tryptophan-indole metabolism to exert anti-AS effects.

Next, targeted metabolomics quantified tryptophan metabolites across three routes: the kynurenine, serotonin and indole pathway (Figure S4). HFD feeding abnormally diverted tryptophan toward the kynurenine pathway while suppressing indole derivatives production. GL treatment broadly restored the levels of various indole derivatives in both feces and plasma, and ILA emerged as the most prominently elevated metabolite (Figure 5F,G). Notably, the plasma concentration of ILA (≈2 μM) matched or exceeded its fecal level, suggesting efficient absorption and systemic bioavailability. In contrast, serotonin pathway metabolites remained largely unaffected. These targeted metabolomic findings were consistent with the untargeted metabolomics data. Together, these results demonstrate that GL redirects tryptophan metabolism from the detrimental kynurenine pathway to the production of indole derivatives, with ILA as the most prominently elevated metabolite in plasma.

To determine whether GL directly promotes ILA production, we performed in vitro anaerobic incubations using fecal microbiota and representative ILA-producing strains (*Bifidobacterium longum* and *Lactobacillus acidophilus*). As shown in Figure S4B–D, GL treatment significantly elevated ILA concentrations in the culture supernatants of both fecal microbiota and mono-cultures. Moreover, GL upregulated the mRNA expression of the key ILA biosynthetic enzymes-aromatic lactate dehydrogenase (*aldh*) in *B. longum* and its functional homolog (*fldh*) in *L. acidophilus* (Figure S4E). These results indicate that GL directly enhances ILA production by gut microbiota, at least in part through transcriptional upregulation of key enzymes in the tryptophan-ILA metabolic pathway.

### 2.6. ILA Administration Recapitulated the Anti-AS Effects of GL in an AhR-Dependent Manner

**Figure 6:**
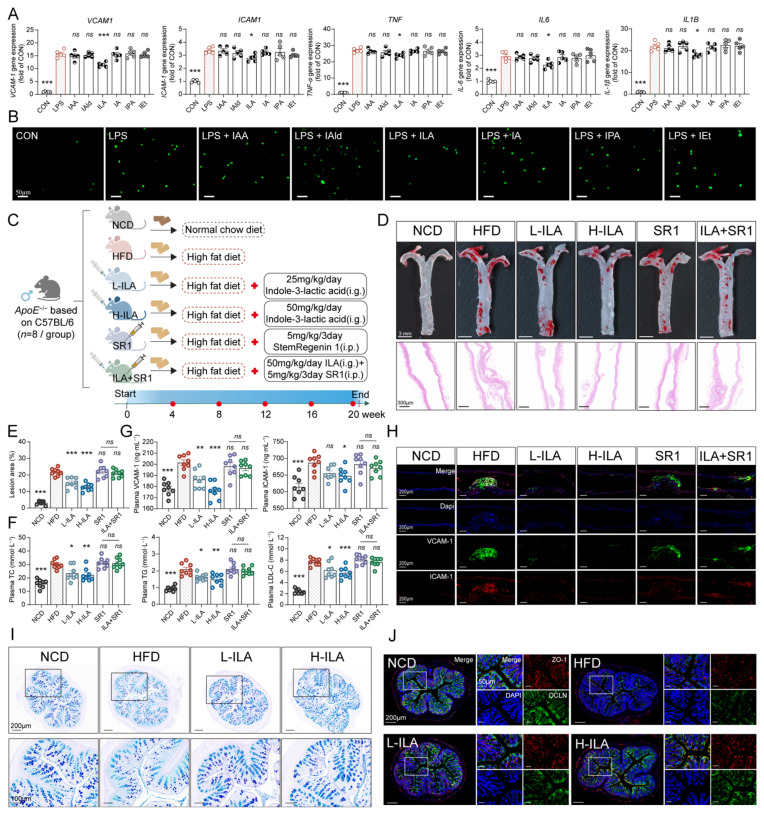
ILA administration recapitulated the anti-AS effects of GL in an AhR-dependent manner. (**A**) The mRNA expression levels of *ICAM1*, *VCAM1*, *TNF*, *IL6* and *IL1B* in HAECs as determined by qRT-PCR (*n* = 5); (**B**) The adhesion of THP-1 monocytes to HAECs was assessed by fluorescence microscopy (scale bars: 50 µm); (**C**) Schematic diagram of experimental design for ILA administration; (**D**) Representative images of oil red O-stained (top, scale bars: 3 mm) and H&E-stained (bottom, scale bars: 300 μm) longitudinal aorta; (**E**) Quantification (%) of oil red O-stained longitudinal aorta (*n* = 8); (**F**) The levels of TC, TG, and LDL-C in plasma (*n* = 8); (**G**) The levels of ICAM-1 and VCAM-1 in plasma (*n* = 8); (**H**) Representative images of immunofluorescence staining for VCAM-1 (green) and ICAM-1 (red) with DAPI counterstaining for nuclei (blue) in the aorta (scale bars: 200 μm); (**I**) Representative images of colonic goblet cells using AB-PAS staining (scale bars: 200 μm); (**J**) Representative immunofluorescence images for ZO-1 (red) and OCLN (green) with DAPI counterstaining for nuclei (blue) in colonic tissue of *ApoE*^−/−^ mice. Data are presented as means ± SEM, * *p* < 0.05, ** *p* < 0.01, *** *p* < 0.001, ns: No significant difference, using ANOVA.

To investigate whether the alleviation of endothelial adhesion and plaque deposition was mediated by these intestinal indole metabolites, we screened a panel of GL-upregulated indole metabolites, including IAA, indole-3-carboxaldehyde (IAld), ILA, indole-3-acrylic acid (IA), IPA and tryptophol (IEt) on HAECs. The in vitro treatment doses were strictly calibrated to match their physiological peak plasma concentrations, as determined by our targeted metabolomics. Under these physiological conditions, only ILA (at 2 μM) successfully suppressed the LPS-induced transcription of adhesion molecules and pro-inflammatory cytokines (Figure 6A). Functionally, ILA prevented the adhesion of THP-1 monocytes to activated HAECs (Figure 6B), identifying ILA as the principal metabolite for mitigating endothelial adhesion and inflammation at physiological blood concentrations.

Subsequently, ILA was administered to HFD-fed *ApoE*^−/−^ mice to evaluate its in vivo anti-AS effects (Figure 6C). Compared to the HFD group, ILA treatment effectively attenuated atherosclerotic plaque formation (Figure 6D,E), improved systemic lipid homeostasis (Figure 6F), and reduced endothelial adhesion molecule expression both in circulation and in situ (Figure 6G,H). ILA is regarded as the principal ligand of AhR, capable of activating the AhR pathway to regulate host immunity and metabolism. We found that the AhR inhibitor StemRegenin 1 (SR1) completely abolished the anti-AS effects mediated by ILA, revealing that ILA exerted its vascular protective effects essentially via AhR signaling.

To determine the upstream origin of this systemic protection, we evaluated intestinal barrier integrity. ILA treatment successfully reversed the HFD-induced depletion of goblet cells and mucin (Figure 6I and Figure S5A) and restored levels of colonic tight junction proteins (ZO-1 and OCLN) (Figure 6J). Consequently, ILA attenuated systemic endotoxemia, as evidenced by significantly reduced plasma LPS levels (Figure S5B).

Collectively, these results demonstrate that ILA is the primary active metabolite responsible for the therapeutic benefits of GL. ILA provides dual protection against AS by reinforcing intestinal barrier integrity and directly inhibiting vascular endothelial activation through an AhR-dependent pathway.

### 2.7. ILA-Activated AhR Interacted with p65 to Suppress VCAM-1/ICAM-1 Transcription

**Figure 7:**
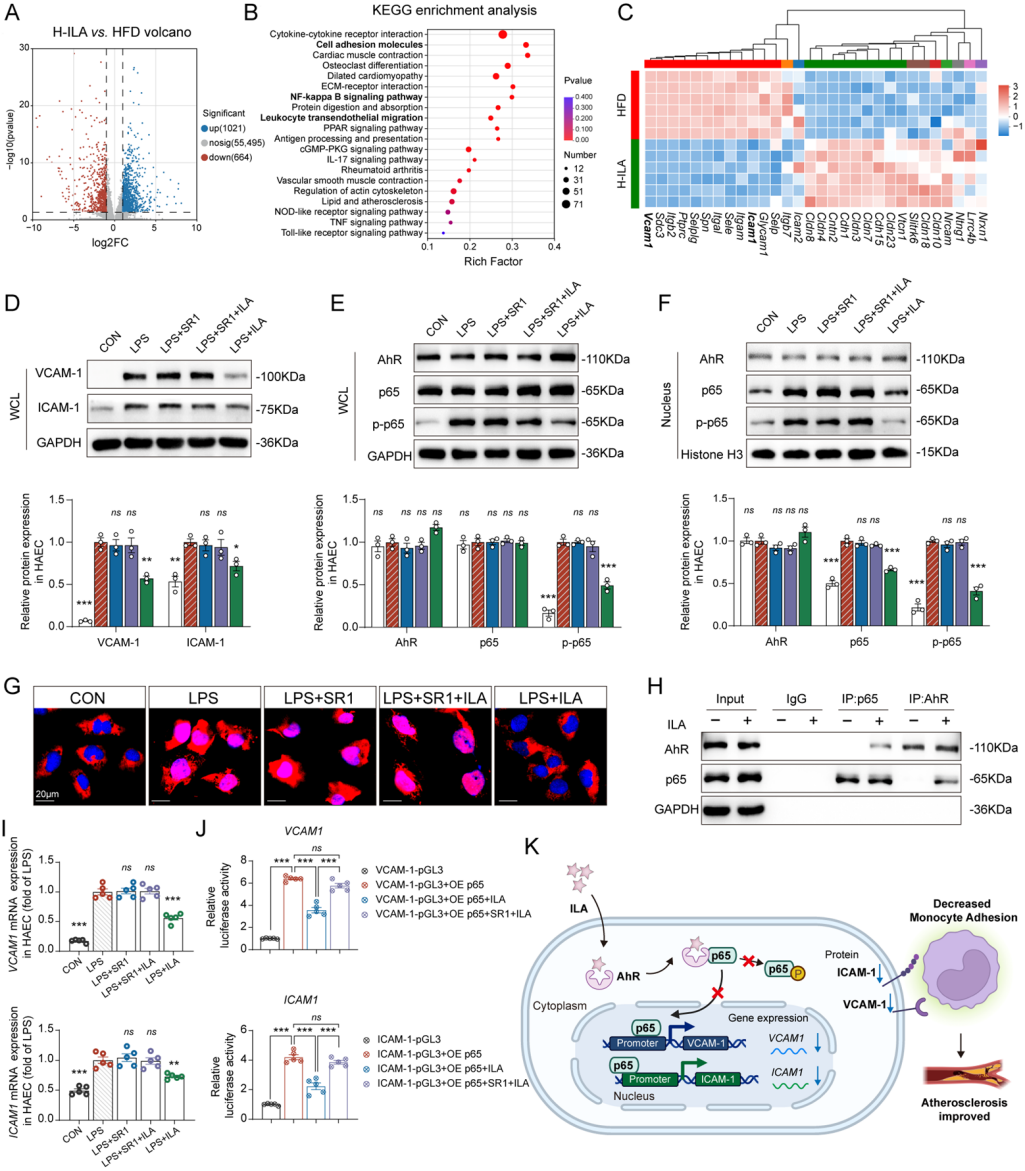
ILA-activated AhR binds p65 to suppress VCAM-1 transcription. (**A**) Volcano plots showed the DEG between H-ILA and HFD group; (**B**) KEGG bubble diagram of enrichment analysis in H-ILA vs. HFD group; (**C**) Cluster heatmap showed the DEGs between H-ILA and HFD group; (**D**) Western blot analysis and quantification of VCAM-1 and ICAM-1 protein expression in WCL (*n* = 3); (**E**) Western blot analysis and quantification of AhR, p65 and p-p65 protein expression in WCL (*n* = 3); (**F**) Western blot analysis and quantification of AhR, p65 and p-p65 protein expression in nucleus (*n* = 3); (**G**) Immunofluorescence analysis of p65 (red) with DAPI counterstaining for cell nuclei (blue) in HAECs (scale bar, 20 μm); (**H**) Representative immunoprecipitation assays showing the interaction between AhR and p65 in HAECs; (**I**) The mRNA expression levels of *VCAM1* and *ICAM1* in HAECs (*n* = 5); (**J**) The relative luciferase activity of *VCAM1* and *ICAM1* under different conditions (*n* = 5); (**K**) Schematic model of the ILA-AhR-p65 molecular crosstalk and downstream signaling pathway. Data are presented as means ± SEM, * *p* < 0.05, ** *p* < 0.01, *** *p* < 0.001, ns: No significant difference, using ANOVA.

To elucidate the molecular mechanisms by which ILA attenuates AS, we first performed RNA-seq on aortic tissues from HFD-fed and ILA-treated mice. Compared to the HFD group, ILA was found to regulate a substantial number of differentially expressed genes (DEGs) (Figure 7A). KEGG analysis revealed that these DEGs were prominently enriched in pathways associated with vascular inflammation and endothelial activation, including the cell adhesion molecules, NF-κB signaling pathway, and leukocyte transendothelial migration (Figure 7B). Given that endothelial–leukocyte adhesion represents the critical initiating event in AS pathogenesis, we focused cluster analysis on the cell adhesion molecules pathway. The result demonstrated that ILA broadly reversed the HFD-induced dysregulation of multiple adhesion-associated genes, including *Vcam1*, *Icam1*, *Sele*, *Selp*, and *Itgam* (Figure 7C). Among this cluster, *Vcam1* and *Icam1* were pinpointed as the central downstream effectors for further validation, as they are the predominant NF-κB driven molecules orchestrating intimal monocyte and macrophage infiltration.

To validate these in vivo transcriptomic findings and explore the underlying regulatory dynamics, we performed in vitro experiments using LPS-stimulated HAECs. Consistent with the transcriptional data, Western blot analysis of whole-cell lysates (WCL) confirmed that ILA profoundly suppressed the LPS-induced protein expression of VCAM-1 and ICAM-1 (Figure 7D). Given that VCAM-1 and ICAM-1 are classical downstream targets of the NF-κB p65 subunit, and ILA is a well-characterized AhR ligand, we next investigated the potential crosstalk between AhR and p65. As shown in Figure 7E, LPS stimulation increased the p65 phosphorylation without affecting total p65 levels in WCL. ILA treatment successfully upregulated AhR expression and attenuated the LPS-induced increase in p65 phosphorylation. To further characterize this AhR activation, we examined its nuclear translocation. Immunofluorescence staining (Figure S6) revealed that despite the overall upregulation, ILA did not induce a significant nuclear translocation of AhR, but remained predominantly in the cytoplasm, suggesting that ILA-activated AhR might exert its regulatory effects through a non-canonical pathway. Since the transcriptional activity of p65 strictly depends on its nuclear translocation, we subsequently analyzed nuclear protein fractions. Compared to the control, LPS induced a significant nuclear accumulation of p65 and p-p65, while ILA effectively inhibited this nuclear translocation (Figure 7F). Furthermore, immunofluorescence staining confirmed that ILA restricted p65 to the cytoplasm (Figure 7G). Crucially, the AhR antagonist SR1 completely abrogated the aforementioned effects of ILA on the phosphorylation and the nuclear translocation of p65. Collectively, these results indicated that ILA inhibited LPS-induced p65 activation and nuclear entry in an AhR-dependent manner.

Then, co-immunoprecipitation (Co-IP) assay in HAECs was employed to elucidate the regulatory relationship between AhR and p65 and explore whether ILA exerts its anti-inflammatory effects via their direct interaction. The results demonstrated that AhR could directly bind to the p65 subunit upon ILA stimulation (Figure 7H), inhibiting the nuclear translocation of p65 and downstream gene expression of *VCAM1* and *ICAM1* (Figure 7I). To further confirm the interaction of AhR-p65 altered promoter activities of *VCAM1* and *ICAM1*, the promoter regions of *VCAM1* or *ICAM1* genes were cloned into the pGL3-Basic luciferase vector and co-transfected with a p65 overexpression plasmid in HEK-293T cells. The results revealed that ILA treatment significantly suppressed the p65-driven promoter activities of both *VCAM1* and *ICAM1*. However, this transcriptional repression was abolished after the blockade of AhR signaling (Figure 7J). Collectively, these findings suggest that ILA ameliorates endothelial adhesion dysfunction and inflammation by targeting the AhR-p65 axis, where ILA-activated AhR directly binds p65 in the cytoplasm, exerting a transcriptional inhibition effect on atherogenic adhesion molecules to mitigate AS (Figure 7K).

## 3. Discussion

The gut microbiota plays vital roles in the pathogenesis of AS; thus, microbial therapeutics may represent a promising strategy for AS patients in the future. However, existing studies still lack precise functionality of the specific bacteria involved and the underlying mechanisms. In this study, we found that GL extracted from licorice exerted excellent anti-AS effects by remodeling the gut microbiota; in particular, we identified the marked enrichment of tryptophan-metabolizing bacteria. Further analysis demonstrated that a novel microbiota–vascular axis mediated the anti-AS effects of GL. Specifically, the increased levels of bacterial-derived ILA effectively suppressed vascular endothelial adhesion and inflammation by promoting a non-canonical AhR-p65 interaction mechanism, thus reducing the AS plaques and lipid deposits.

Licorice (*Glycyrrhiza glabra* L.) is an important “medicine food homology” plant and has been used for centuries in traditional Chinese medicines because of its excellent therapeutic properties and safety. GL, a natural triterpenoid glycoside extracted from the roots of licorice, exhibits an extensive range of pharmacological activities, including anti-inflammation, anti-oxidation, and liver protection, thus suggesting its potential for treating metabolism-related diseases triggered by inflammation and oxidative stress, such as AS [34]. Previous researchers have reported that GL effectively reduced the intimal thickness of the carotid artery and the formation of plaques in both rats with diabetes mellitus and HFD-fed *ApoE*^−/−^ mice by improving endothelial dysfunction and glucolipid metabolism [30,35]. However, the drug concentration of orally administered GL in the blood is much lower than that achieved by injection because of its low bioavailability. In this study, we revealed that the plasma *C_max_* of oral GL (100 mg/kg) did not effectively improve the adhesion of endothelial cells in vitro; this contradicted our in vivo results in which oral GL demonstrated a significantly superior anti-AS effect when compared to GL injection. This finding suggests that the mechanism of oral GL is not limited to its direct effects on the AS-related signaling pathway and molecular targets by absorption into the bloodstream. Due to its poor intestinal absorption, oral GL accumulates in relatively high concentrations in the intestine, where it facilitates interaction with the gut microbiota and intestinal epithelial cells to indirectly manifest probiotic functions. In the present study, the combination of antibiotic application with an FMT strategy revealed that the anti-atherosclerotic effect of GL is mediated by the gut microbiota, at least in part. This finding motivated us to further investigate the effects of GL on modulating the gut microbiota.

Dysbiosis of the gut microbiota induced by Western diets has been widely shown to exert significant influence on lipid metabolism and the inflammatory response. A cross-sectional study of 8973 participants from the SCAPIS cohort provided robust evidence of an association in the gut microbiota composition characterized by an increased abundance of *Streptococcus* spp. with coronary atherosclerosis and systemic inflammation markers [36]. In addition, *Desulfovibrio desulfuricans* was shown to aggravate AS by enhancing intestinal permeability and endothelial TLR4/NF-κB pathway in *ApoE*^−/−^ mice [37]. In contrast, *Flavonifractor plautii* was shown to protect against elevated arterial stiffness through the suppression of matrix metalloproteinase-2 activation [38]. Our results of fecal metagenomic sequencing showed that GL efficiently modulated the diversity, structure and composition of the gut microbiota at different taxonomical levels. At the phylum level, we found that GL restored the *Bacillota* (*Firmicutes*)/*Bacteroidetes* ratio disrupted by the HFD, which has been considered as an important marker of obesity and abnormal lipid metabolism in many studies [39,40]. At the genus level, GL markedly suppressed the abundance of LPS-producing pathobionts, particularly *Desulfovibrio*. By reducing this endotoxin source and effectively repairing the intestinal mucus barrier and tight junctions, GL markedly limited intestinal leakage of LPS, thereby eliminating a critical trigger of endothelial activation. Beyond this barrier-protective effect, we identified the novel capability of the reshaped gut microbiota to metabolize tryptophan towards the robust production of indole derivative, as determined by fecal/plasma untargeted and targeted metabolomics analysis.

Tryptophan metabolism primarily involves the kynurenine, 5-hydroxytryptamine and indole pathways. The gut microbiota convert an amount of tryptophan into indole and its derivatives, such as IAA, IPA, ILA, and IEt [41]. In HFD mice, LPS stimulated colonic immune responses to upregulate the indoleamine 2,3-dioxygenase 1 (IDO1)-mediated kynurenine pathway, leading to tryptophan depletion and kynurenine accumulation in the circulation [42]. Our current results showed that GL treatment effectively blunted this host-driven metabolic shift and restored the levels of various indole derivatives. Mechanistically, GL treatment significantly increased the abundance of specific symbiotic species particularly *Bacteroides acidifaciens*, *Bacteroides ovatus*, *Bifidobacterium pseudolongum*, *Bifidobacterium breve*, *Lactobacillus johnsonii*, and *Lactobacillus intestinalis*. These highly enriched bacteria have been found to convert tryptophan to ILA via the aromatic amino acid aminotransferase (ArAT) and an indolelactic acid dehydrogenase (ILDH) [43]. Interestingly, while multiple indoles were abundant in feces, ILA emerged as the most prominently elevated circulating metabolite in plasma, suggesting efficient absorption and systemic bioavailability. Notably, the in vitro therapeutic efficacy of ILA was strictly validated at its physiological peak plasma concentration (≈2 μM), confirming its role as the primary active metabolite mitigating endothelial inflammation.

ILA has been increasingly recognized as a crucial metabolite capable of maintaining mucosal and systemic homeostasis, primarily functioning as an endogenous ligand for the AhR. For instance, the amelioration of colitis can depend on the activation of the AhR signaling pathway, mediated by ILA primarily derived from *Lactobacillus* [44]. Beyond receptor activation, the anti-inflammatory efficacy of ILA is mechanistically tied to the suppression of classic inflammatory cascades by downregulating the NF-κB signaling pathway via AhR [45]. Crucially, the protective effect of the ILA-AhR axis extends beyond the intestinal epithelium. Emerging evidence indicates that ILA also reinforces the gut vascular barrier (GVB) against ischemia/reperfusion injury [46]. However, whether ILA directly affects vascular endothelium remained unknown. Building on these mechanistic foundations, we investigated whether ILA could exert distant atheroprotective effects on vascular endothelium.

Our study demonstrated that ILA effectively inhibited LPS-induced vascular endothelial activation and monocyte adhesion, whereas the administration of an AhR inhibitor (SR1) abrogated these atheroprotective effects both in vivo and in vitro. The biological outcome of AhR signaling is largely determined by the nature of its bound ligands. Classically, upon ligand binding, activated AhR translocates into the nucleus, dimerizes with the ARNT, and transcriptionally regulates target genes. However, an emerging paradigm of non-canonical AhR signaling highlights its alternative capacity to interact with proteins other than ARNT [47]. It has been reported that AhR interacts with the NF-κB subunit p65 [48]. Our current findings uncover this exact non-canonical mechanism within the vascular endothelium. Using Co-IP and dual-luciferase reporter assays, we demonstrated that ILA-activated AhR directly interacts with the NF-κB subunit p65 in the cytoplasm. This interaction sequesters p65 in the cytosol, thereby inhibiting its nuclear translocation. As a result, the p65-mediated transcriptional activation of *VCAM1* and *ICAM1* is significantly suppressed. These findings identify a novel ILA-AhR-p65 interaction axis, revealing the molecular mechanism by which gut-derived ILA attenuates endothelial activation and subsequent monocyte infiltration.

To better contextualize our findings, it is instructive to compare the mechanism of GL with those of other anti-AS triterpenoids. Triterpenoids are broadly categorized as free aglycones or glycosylated derivatives (saponins) [49]. Although they generally exhibit poor oral bioavailability, the prevailing view is that the atheroprotective effects are mediated by a small fraction of systemically absorbed compounds that exert direct pharmacological actions. For example, ganoderic acids directly attenuate macrophage-mediated inflammation by suppressing TLR4/NF-κB signaling [50], and maslinic acid modulates Nrf2-dependent pathways to alleviate endothelial dysfunction [51]. Similarly, *Panax notoginseng* saponins reprogram macrophage metabolism by modulating sphingolipid metabolism, thereby rebalancing macrophage polarization [52]. Ginsenoside Rb1 inhibits VSMC proliferation and foam cell formation by suppressing c-Jun/AP-1-dependent LOX-1 transcription and activating autophagy [53]. Our study demonstrates that GL acts through a novel microbiota–vascular axis that distinguishes from other triterpenoids. Rather than relying on systemic absorption, GL acts locally to repair the intestinal barrier and reprogram microbial tryptophan metabolism toward ILA production. The resulting metabolite then activates AhR to physically sequester p65 in the cytoplasm of arterial endothelial cells, thereby suppressing VCAM-1/ICAM-1 transcription.

This study has several limitations. First, although our results identified the microbial ILA-AhR-p65 axis as a major atheroprotective mechanism of GL, the concurrent direct pharmacological effects on the host cannot be entirely ruled out. Second, the evidence that the gut microbiota participated in the anti-AS effects of GL is based on the AB-treated pseudo germ-free *ApoE*^−/−^ mice. The use of gnotobiotic mice would avoid the possible effects of AB on gut barrier and further strengthen causality of our findings. Finally, our mechanistic insights rely heavily on murine models. Given the species-specific differences in gut microbiota composition and AhR ligand affinities, the endothelial protection mediated by ILA needs to be further validated in clinical cohorts.

## 4. Materials and Methods

### 4.1. Materials

The reagents and antibodies applied in this study are shown in Supplementary Table S1.

### 4.2. Mice and Treatments

Healthy 6-week-old male *ApoE*^−/−^ mice from a C57BL/6J background, weighing 20–22 g, were purchased from SPF (Beijing) Biotechnology Co., Ltd. (Beijing, China). and housed in a specific pathogen-free facility under a 12 h light/dark cycle with free access to food and water. *ApoE*^−/−^ mice were fed an HFD to induce AS after one week of acclimation. All animal experiments (experiments 1–4) were conducted in accordance with rules set by the Laboratories Institutional Animal Care and Use Committee (IACUC) of the Chinese Academy of Medical Sciences and Peking Union Medical College (Approval number: 00004122). The intervention schedule lasted for 20 weeks, and the body weight of each mouse was monitored weekly. At the end of the drug intervention, all mice were deprived of food and water for 12 h for the following sample collection. Fresh feces were frozen in liquid nitrogen and then stored at −80 °C for gut microbiota and bacteria-derived metabolite analysis. Blood samples were collected in the EDTA-2K-pretreated collection tube and centrifuged at 3500 rpm (15 min, 4 °C) for plasma separation. Mice were sacrificed via cervical dislocation, and tissues (aorta, colon, kidney) were either immersed in 4% paraformaldehyde for histological staining or stored at −80 °C for further analysis.

Experiment 1. The efficacy of GL on the amelioration of AS. *ApoE*^−/−^ mice were randomly divided into five groups (*n* = 8/group): (1) normal chow diet (NCD) group, (2) high-fat diet (HFD) group, (3) LG group (HFD + 50 mg/kg/day GL, by gavage), (4) HG group (HFD + 100 mg/kg/day GL, by gavage) and (5) GL-ip group (HFD + 30 mg/kg/day GL, intraperitoneally).

Experiment 2. Gut Microbiota Depletion. The antibiotic cocktail, a treatment regimen that has been shown to effectively deplete the gut microbiota, consisted of vancomycin (50 mg/kg), neomycin (100 mg/kg), metronidazole (100 mg/kg), and amphotericin (1 mg/kg). *ApoE*^−/−^ mice were divided into four groups (*n* = 8/group): (1) HFD group, (2) AB group (HFD + antibiotic cocktail, by gavage), (3) HG group (HFD + 100 mg/kg/day GL, by gavage) and (4) HG +AB group (HFD + antibiotic cocktail + 100 mg/kg/day GL, by gavage).

Experiment 3. Fecal Microbiota Transplantation (FMT) [54]. Fresh feces from the HFD or HG groups were collected (500 mg), resuspended in phosphate-buffered saline (PBS, 5 mL) and centrifuged at 100 rpm for 1 min at 4 °C. The precipitate was washed three times with PBS (5 mL per wash). All supernatants were collected and centrifuged at 5000 rpm for 20 min, after which the resulting fecal bacterial precipitate was resuspended in PBS (2 mL) and administered to antibiotic-pretreated recipient mice by oral gavage (200 μL, twice weekly, *n* = 8/group): (1) HFD-FMT group (AB-pretreated HFD recipient *ApoE*^−/−^ mice treated with fecal bacteria of HFD donor mice) and (2) HG-FMT group (AB-pretreated HFD recipient *ApoE*^−/−^ mice treated with fecal bacteria of HG donor mice).

Experiment 4. The efficacy of ILA on the amelioration of AS. Groups were as follows (*n* = 8/group): (1) NCD group, (2) HFD group, (3) L-ILA group (HFD + 25 mg/kg/day ILA, by gavage), (4) H-ILA group (HFD + 50 mg/kg/day ILA, by oral gavage), (5) Stemregenin 1 (SR1) group (HFD + 5 mg/kg/3 day SR1, intraperitoneally), and (6) ILA + SR1 group (HFD + 50 mg/kg/day ILA + 5 mg/kg/3 day SR1).

### 4.3. Biochemical Assays

The plasma levels of total cholesterol (TC), triglycerides (TG), low-density lipoprotein (LDL-C), potassium (K^+^), sodium (Na^+^) were quantified using commercially available kits with an automatic biochemical analyzer (TOSHIBA, Tokyo, Japan), following the manufacturer’s protocols. The levels of oxidized low-density lipoprotein (ox-LDL), intercellular adhesion molecule-1 (ICAM-1), vascular cell adhesion molecule-1 (VCAM-1), monocyte chemoattractant protein-1 (MCP-1), aldosterome, renin, and LPS in plasma were assessed using corresponding ELISA kits. Plasma cytokines, including tumor necrosis factor-alpha (TNF-α), IL-6, IL-1β, IL-17, IL-4, and IL-10, were measured in batch analyses using a Bio-Plex 200 Bioanalyzer (Bio-Rad, Hercules, CA, USA), in accordance with the manufacturer’s instructions.

### 4.4. Histology Staining

Oil Red O or H&E staining of aorta: Specimens of aorta were dissected from the proximal ascending aorta to the iliac bifurcation and fixed in 4% paraformaldehyde. After removing adventitial fat, the mouse aortas were processed for staining. The kidney was also removed and stained by H&E reagents. Digital images were analyzed using ImageJ software (v.1.53a, NIH, Bethesda, MD, USA), and the extent of the lesion area was expressed as the percentage of the total aortic area covered by the lesions.

AB-PAS staining: Colon tissue specimens were embedded in paraffin, sectioned into 4 μm thick slices, and stained by AB-PAS reagents to determine the number of goblet cells.

During the experimental period, the body weight, stool consistency, and fecal occult blood were recorded daily. At the end of the experiment, all mice were executed by inhalation of isoflurane, followed by the rapid collection of blood, feces, cecal contents, and colonic tissues for subsequent analyses.

### 4.5. Immunofluorescence Staining

Paraffin sections of aorta were deparaffinized, rehydrated, and subjected to antigen retrieval using EDTA buffer. Subsequently, sections were sequentially processed by first incubating with anti-VCAM-1 antibody overnight at 4 °C, followed by HRP-conjugated goat anti-rabbit IgG and IF488-Tyramide amplification. Next, we performed microwave stripping in EDTA buffer to remove antibody complexes. Sections were then incubated with anti-ICAM-1 rabbit antibody overnight at 4 °C, followed by HRP-conjugated goat anti-rabbit IgG and CY3-Tyramide amplification. Finally, sections were counterstained with DAPI. Colonic samples were incubated with anti-ZO-1 and anti-OCLN antibodies. After washing, the slides were incubated with CY3-conjugated goat anti-rabbit secondary antibody or FITC-conjugated goat anti-mouse secondary antibody, and counterstained with DAPI.

### 4.6. Cell Culture and Treatment

HAEC (RRID: CVCL_C0EQ) and THP-1 (RRID: CVCL_0006) cell lines were obtained from the Cell Resource Center, Peking Union Medical College (which is the headquarter of National Infrastructure of Cell Line Resource, NSTI) and checked free of mycoplasma contamination. HAECs were cultured in endothelial cell medium (ECM) supplemented with 10% heat-inactivated fetal bovine serum (FBS), 1% endothelial cell growth supplement (ECGS), and 1% penicillin/streptomycin solution (P/S). THP-1 cells were maintained in RPMI 1640 medium supplemented with 10% heat-inactivated FBS and 1% P/S solution. All cells were incubated at 37 °C in an atmosphere of 5% CO_2_ and 90% relative humidity.

HAECs were assigned to five experimental groups: (1) untreated control (CON); (2) LPS (0.1 μg/mL); (3) LPS (0.1 μg/mL) + SR1 (1 μM); (4) LPS (0.1 μg/mL) + SR1 (1 μM) + ILA (2 μM); and (5) LPS (0.1 μg/mL) + ILA (2 μM). For AhR blockade, cells were pretreated with SR1 for 1 h before LPS stimulation, whereas ILA was co-administered with LPS for 24 h. After the 24 h treatment, cells from all groups were collected for whole-cell and nuclear immunoblotting.

### 4.7. In Vitro Metabolite Screening and Monocyte Adhesion Assay

For in vitro assays, HAECs (2 × 10^5^ cells/well) in 12-well plates were treated with 0.1 μg/mL LPS alone or together with indole metabolites for 24 h. Doses of each metabolite were adjusted to their respective peak plasma concentrations in GL-treated mice determined by targeted tryptophan metabolomics: indole-3-acetic acid (IAA, 0.21 μM), indole-3-carboxaldehyde (IAld, 0.12 μM), indole-3-acrylic acid (IA, 0.0067 μM), indole-3-propionic acid (IPA, 0.15 μM), tryptophol (IEt, 0.025 μM), and indole-3-lactic acid (ILA, 2.20 μM).

After 24 h of treatment, HAECs were either harvested for gene expression analysis or used for a monocyte adhesion assay. For the adhesion assay, THP-1 cells were labeled with 2 μM calcein-AM for 30 min, resuspended at 1 × 10^5^ cells/mL, and co-incubated with the treated HAECs for 120 min. Non-adherent cells were removed by three gentle washes with PBS containing 1% FBS. The remaining adherent THP-1 cells were fixed with 4% paraformaldehyde, and fluorescent images were captured with a fluorescence microscope (Zeiss, Oberkochen, Germany).

### 4.8. Fecal Metagenomic Sequencing and Analysis

Bacterial DNA from feces samples was extracted with the FastPure Stool DNA Isolation Kit (MJYH, Shanghai, China). The integrity of extracted DNA was examined by 1% agarose gel electrophoresis. The DNA concentration and purity were determined by TBS-380 (Turner BioSystems, Sunnyvale, CA, USA) and NanoDrop200 fluorometers (Thermo Fisher Scientific, Waltham, MA, USA), respectively. Then, the DNA was fragmented into 350 bp fractures by using Covaris M220 (Gene Company, Beijing, China). After selecting samples that meet the quality standards, construct a sequencing library. Use Illumina HiSeq high-throughput sequencing platform to sequence and analyze the gut microbiome genome. The raw sequencing data were quality-checked and filtered on the Majorbio Cloud Platform (https://www.majorbio.com, accessed on 6 April 2025).

### 4.9. Fecal Untargeted Metabolomics Analysis

The untargeted metabolomics was conducted by Thermo UHPLC-Q Exactive HF-X system (Thermo Fisher Scientific, Waltham, MA, USA) equipped with an ACQUITY HSS T3 column (Waters Corporation, Milford, MA, USA) as described in our previous studies, respectively [54,55]. Data processing and analysis were performed on the Majorbio cloud platform (https://cloud.majorbio.com, accessed on 22 May 2025).

### 4.10. RNA-Sequencing Analysis

Genome-wide gene expression analysis was performed on aortic tissues from the HFD group and H-ILA group (*n* = 6). RNA extraction, library preparation, sequencing, quality control, and read mapping were conducted by Shanghai Majorbio Bio-pharm Technology Co., Ltd. (Shanghai, China). The differentially expressed genes (DEGs) were identified based on *p* < 0.05 and a fold change ≥ 2. Differential expression analysis was carried out using DESeq2 for samples with biological replicates. KEGG enrichment analyses were performed using a hypergeometric distribution algorithm to identify significantly enriched functional categories. Cluster analysis was conducted to compare gene expression profiles between HFD and ILA-treated *ApoE*^−/−^ mice.

### 4.11. Targeted Metabolomics Analysis of Tryptophan Metabolites

Sample Preparation: For fecal samples, 25 mg of each sample was homogenized in methanol:water (4:1) by cryo-milling (−10 °C, 50 Hz, 6 min), followed by sonication (40 kHz, 5 °C, 30 min). Then, 100 μL of fecal homogenates or plasma samples were mixed with 10 μL of internal standard (Trp-D5, 4000 ng/mL) and 390 μL of methanol. After vortexing, sonication (40 kHz, 5 °C, 30 min) and centrifugation (12,000 rpm, 4 °C, 15 min), the supernatant was collected and dried under N_2_, before being dissolved in 70 μL of acetonitrile containing 0.1% formic acid for the analysis of tryptophan metabolites.

LC-MS/MS analysis: Quantitative analysis was performed on a Nexera Series LC-40 system coupled with a QTRAP 6500+ mass spectrometer (Sciex, Redwood City, CA, USA) at Majorbio Bio-Pharm Technology Co. Ltd. (Shanghai, China). The separation of metabolites was achieved using an ACQUITY UPLC HSS T3 (2.1 × 150 mm, 1.8 µm) column at 40 °C. The mobile phase comprised water with 0.1% formic acid (solvent A) and acetonitrile with 0.1% formic acid (solvent B), at a flow rate of 1 mL/min through an 18 min gradient elution program. Ionization was performed with the ion source temperature stabilized at 550 °C, utilizing optimized curtain gas and nebulizer gas configurations. Quantitation of the metabolite concentrations was performed utilizing a linear regression standard curve based on the data collected during LC-MS analysis [56].

### 4.12. RNA Extraction and Real-Time qPCR

Total RNA was extracted from tissue using a TRIzol Plus RNA Purification Kit (Invitrogen, Carlsbad, CA, USA). RNA concentrations were measured using a DS-11 Spectrophotometer (Denovix, Wilmington, DE, USA), and 1 μg of purified RNA from each sample was reverse-transcribed into cDNA using the HiFiScript cDNA Synthesis Kit. qPCR was performed on a CFX Connect Real-Time PCR Detection System (Bio-Rad, Hercules, CA, USA) using Ultra SYBR Mix (Low ROX). The results are presented as fold changes relative to *GAPDH* and were calculated using the 2^−ΔΔCT^ method [57]. The primers used for RT-qPCR are listed in Table S2.

### 4.13. Western Blotting

Proteins were extracted from the colon and HAEC cells using radioimmunoprecipitation assay (RIPA) lysis buffer supplemented with protease and phosphatase inhibitors. Protein concentrations were determined with a BCA Protein Quantification Kit (Thermo Fisher Scientific, Waltham, MA, USA). Sodium dodecyl sulfate–polyacrylamide gel electrophoresis (SDS-PAGE) was used to separate the proteins in the samples. The separated proteins were subsequently transferred to a polyvinylidene difluoride (PVDF) membrane and incubated with an antibody specific to the target protein.

### 4.14. Co-IP Assay

After transfection or stimulation, the cells were lysed with NP40 lysis buffer containing 1% phosphatase inhibitor and 1% protease inhibitor for 30 min on ice. The lysates were then centrifuged at 12,000 rpm for 5 min at 4 °C. For immunoprecipitation, the cell lysates were incubated with 2 μg of anti-p65 antibody or 2 μg of anti-AhR antibody overnight at 4 °C with gentle rotation. Normal rabbit IgG was used as a negative control. The next day, 30 μL of Protein A/G magnetic beads were added to each sample and incubated for another 3 h at 4 °C. The beads were collected using a magnetic rack and washed three times with 500 μL of precooled NP-40 lysis buffer. Finally, the agarose bead-antigen-antibody complexes were resuspended in 60 μL of loading buffer, denatured at 100 °C for 5 min, and analyzed by Western blotting.

### 4.15. Dual-Luciferase Reporter Assay

The promoter regions from −2500 bp to +100 bp of *ICAM1* and *VCAM1* were cloned into pGL3-basic plasmid, respectively. HEK-293T cells were seeded into 24-well plates and cultured to 70–80% confluence. For the assay, cells were transfected with the target promoter reporter plasmid (VCAM-1-pGL3 or ICAM-1-pGL3), the pRL-TK control plasmid, and the p65 overexpression plasmid (OE p65) using PEI reagent (plasmid:PEI = 0.55 μg:1.65 μL per well). The corresponding drug or PBS was added to each group after 24 h of transfection. Finally, cells were lysed, and the luciferase activity was detected using a Dual-Luciferase Reporter Assay System. The relative luciferase activity was calculated as the ratio of Firefly luciferase activity to Renilla luciferase activity.

### 4.16. Statistical Analysis

All statistical analysis was performed using GraphPad Prism version 8.0 (GraphPad Software, San Diego, CA, USA) with data presented as mean ± standard error of the mean (SEM). Statistical significance was defined as *p* < 0.05 after adjustment for multiple comparisons where applicable. Statistical tests were selected based on appropriate assumptions with respect to data distribution and variance characteristics. Two-group comparisons employed unpaired two-tailed Student’s *t*-test (parametric) or Mann–Whitney *U* test (non-parametric), while multi-group analyses were performed with one-way ANOVA with Dunnett’s post hoc test (parametric) or Kruskal–Wallis with Dunn’s correction (non-parametric). Spearman’s correlation was used to test for relationships between bacterial abundances, metabolites and disease markers. RDA was used to identify microbiome-phenotype associations. The sample size (*n*) is stated in the figure legends to indicate the number of biologically independent replicates used for statistical analyses. Animals were excluded when an objective experimental failure was observed. Values detected by the two folds of standard deviation were discarded.

## 5. Conclusions

In conclusion, our findings demonstrate that oral GL exerted significant anti-AS effects by modulating the gut microbiota and associated metabolic pathways. Specifically, GL enriched tryptophan-metabolizing bacteria, resulting in the increased production of the microbial metabolite ILA, which acts as a ligand of AhR, improving intestinal barrier integrity and alleviating endothelial inflammation. Notably, circulating ILA-activated AhR physically interacted with the NF-κB subunit p65 in the cytoplasm, effectively suppressing the transcription of atherogenic adhesion molecules. These findings highlight the therapeutic potential of targeting the microbiota-derived ILA-AhR-p65 axis, and position GL as a promising candidate for the management of AS.

## Data Availability

All data generated in the current study are available from the corresponding author upon reasonable request.

## Supplementary Materials

The following supporting information can be downloaded at https://www.mdpi.com/article/10.3390/ijms27156694/s1.

## Abbreviations

The following abbreviations are used in this manuscript:

NCD	Normal chow diet
HFD	High-fat diet
GL	Glycyrrhizic acid
AS	Atherosclerosis
TC	Total cholesterol
TG	Triglycerides
LDL-C	Low-density lipoprotein cholesterol
ox-LDL	Oxidized low-density lipoprotein
ICAM-1	Intercelladhesion molecule-1
VCAM-1	Vascular cell adhesion molecules-1
MCP-1	Monocyte chemoattractant protein-1
LPS	Lipopolysaccharide
AB	Antibiotic cocktail
FMT	Fecal microbiota transplantation
TNF-α	Tumor necrosis factor-alpha
Il-1β	Interleukin-1β
Il-6	Interleukin-6
PCoA	Principal coordinate analysis
LEfSe	Linear discriminant analysis effect size
RDA	Redundancy analysis
KEGG	Kyoto Encyclopedia of Genes and Genomes
LDA	Linear discriminant analysis
Zo1	Zonula occludens-1
Ocln	Occludin
Cldn1	Claudin-1
F11r	Junctional adhesion molecule A
MUC	mucin
AhR	Aryl hydrocarbon receptor
NF-κB	Nuclear factor kappa-light-chain-enhancer of activated B cells
IAA	Indole-3-acetic acid
IAld	Indole-3-carboxaldehyde
ILA	Indole-3-lactic acid
IA	Indole-3-acrylic acid
IPA	Indole-3-propionic acid
IEt	Tryptophol
