# Supplementary material for: Glycyrrhizic Acid Alleviates Atherosclerosis in ApoE−/− Mice via Microbial Indole-3-Lactic Acid-Mediated AhR-p65 Interaction in the Endothelium
Source: Int J Mol Sci. 2026 Jul 27;27(15):6694. doi: 10.3390/ijms27156694 (PMC13467423; doi:10.3390/ijms27156694)
Supplement: Supplementary file 1 [file ijms-27-06694-s001.zip › ijms-4366085-supplementary.pdf]

Supplementary Materials for

Glycyrrhizic Acid Alleviates Atherosclerosis in ApoE−/− Mice via Microbial Indole-3-Lactic Acid-Mediated AhR-p65 Interaction in the Endothelium

Haoran Shen [1,†], Shuai Huang [2,†], Zhiyu Wang [1], Sitong Zhou [1], Lulu Huang [3], Hongjuan Zhang [1], Yanxing Han [1], Jiandong Jiang [1,4,*] and Huihui Guo [1,*]

[1] State Key Laboratory of Bioactive Substance and Function of Natural Medicines, Institute of Materia Medica, Chinese Academy of Medical Sciences & Peking Union Medical College, Beijing 100050, China; shenhaoran@imm.ac.cn (H.S.); wangzhiyu@imm.ac.cn (Z.W.); zhousitong@imm.ac.cn (S.Z.); zhjuan@imm.ac.cn (H.Z.); hanyanxing@imm.ac.cn (Y.H.)
[2] Biomedical Innovation Center, Beijing Shijitan Hospital, Capital Medical University, Beijing 100038, China; huangshuai@bjsjth.cn
[3] Hubrecht Institute, Royal Netherlands Academy of Arts and Sciences (KNAW) and University Medical Center Utrecht, 3584 Utrecht, The Netherlands; l.huang@hunrecht.eu
[4] Institute of Medicinal Biotechnology, Chinese Academy of Medical Sciences & Peking Union Medical College, Beijing 100050, China
* Correspondence: jiangjd@imm.ac.cn (J.J.); guohh@imm.ac.cn (H.G.)
† These authors contributed equally to this work.

This file includes:

Supplementary Methods

Figures S1 to S6

Tables S1 to S2

Supplementary Methods

Fecal β-Gus activity

Fecal β-Gus activity was assessed by 4-nitrophenyl-β-d-glucopyranoside (PNPG) assay. In brief, stool homogenate supernatant was quantified using the BCA Protein Assay Kit. The reaction system was consisted of 20 μL of protein, 20 μL of 0.5 mmol/L PNPG, and 80 μL of PBS. The reaction solution was measured at 405 nm after incubation at 37°C for 30 min.

In vitro anaerobic incubation of fecal microbiota

Fresh feces (2 g) were collected from *ApoE*$^{-/-}$ mice and pre-incubated for 60 min in normal saline (feces : saline [w/v] = 1:20) under anaerobic conditions (N_2 : H_2 : CO_2 = 8:1:1, 37 °C) and mixed thoroughly. Then, the intestinal bacterial cultures was obtained by gentle centrifugation (100 rpm, 5min, 4°C). The cultures were then incubated with or without GL (100 μM) for 12 h under anaerobic conditions.

Bacterial strains and mono-culture conditions

Bifidobacterium longum was grown in reinforced clostridial medium (RCM, BNCC, China) under anaerobic conditions (N_2 : H_2 : CO_2 = 8:1:1, 37 °C). *Lactobacillus acidophilus* was grown in de Man-Rogosa-Sharpe broth (MRS, BNCC, China) under the same anaerobic conditions. GL (100 μM) or vehicle control was added to bacterial strains followed by incubation for 12 h at 37 °C.

Sample collection and ILA quantification

After incubation, the bacterial cultures were centrifuged (10,000 rpm, 10 min, 4 °C), and the supernatants were collected to determine the amount of ILA produced by the fecal microbiota and bacterial strains using LC-MS/MS as described in the main Methods section 4.11.

Bacterial RNA extraction and real-time qPCR

Bacteria were collected by centrifugation (10,000 rpm, 10 min, 4 °C) and total RNA was extracted using TRIzol Plus Kit with an initial lysozyme treatment to ensure efficient lysis of the cell wall. Reverse transcription and RT-qPCR were performed as described in Section 4.12. Relative expression levels of *aldh* and *fldh* were normalized to 16S rRNA (using primers listed in Table S2) and calculated by the $2^{-\Delta\Delta Ct}$ method.

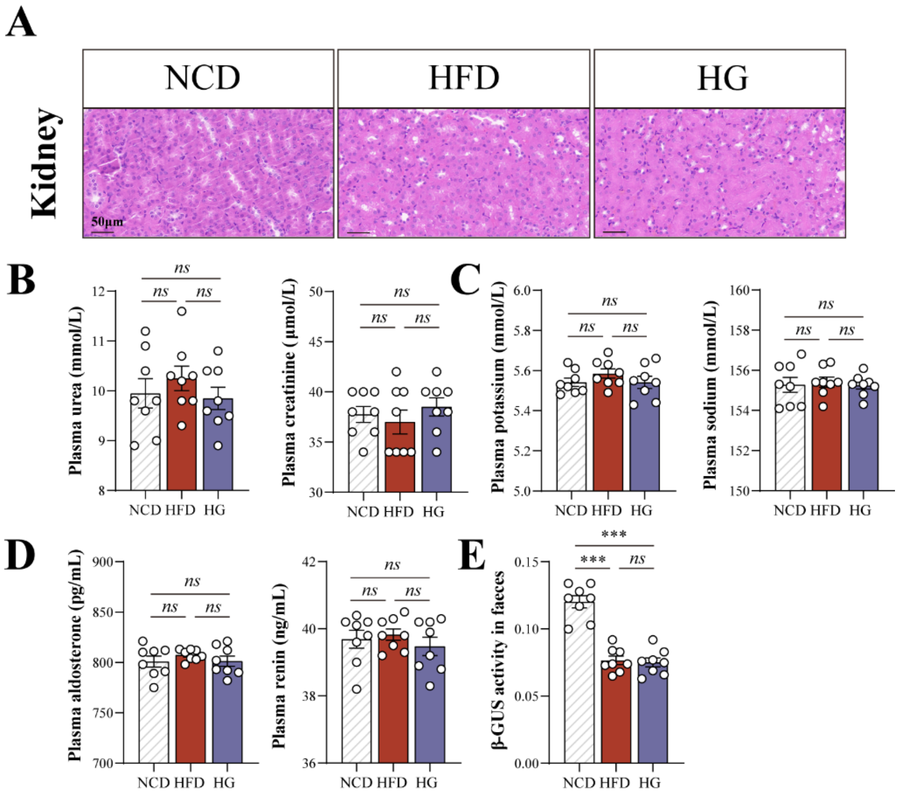

Figure S1. Safety profile of GL *in vivo*. (**A**) Representative images of H&E-stained kidney (scale bars: 50 μm). (**B**) The levels of urea and creatinine in plasma (n = 8). (**C**) The levels of potassium and sodium in plasma (n = 8). (**D**) The levels of plasma aldosterone and renin as determined by ELISA (n = 8). (**E**) β-Gus activity in faeces was detected by PNPG assay (n = 8). Data are presented as means ± SEM, *** $p < 0.001$, ns = non-significant, using ANOVA.

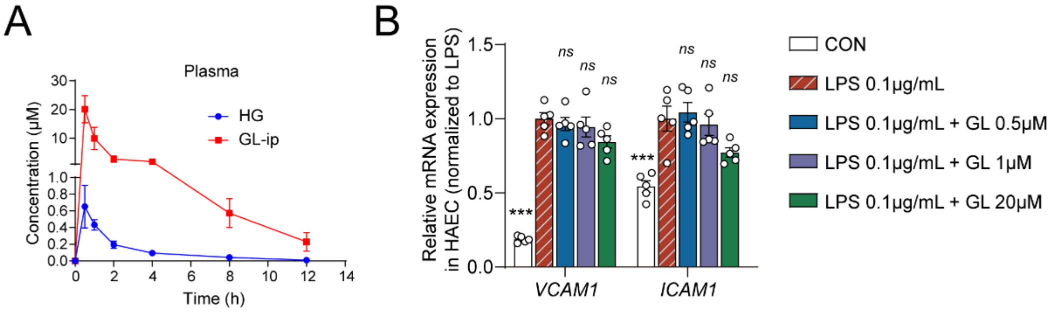

Figure S2. The plasma drug-time curves for oral and intraperitoneal GL. (**A**) Plasma concentration-time profiles of GL (n = 5). (**B**) Relative mRNA level of *VCAM1* and *ICAM1* in HAECs (n = 5).Data are presented as means ± SEM. Statistical significance was assessed using one-way ANOVA. *** $p < 0.001$, ns = non-significant.

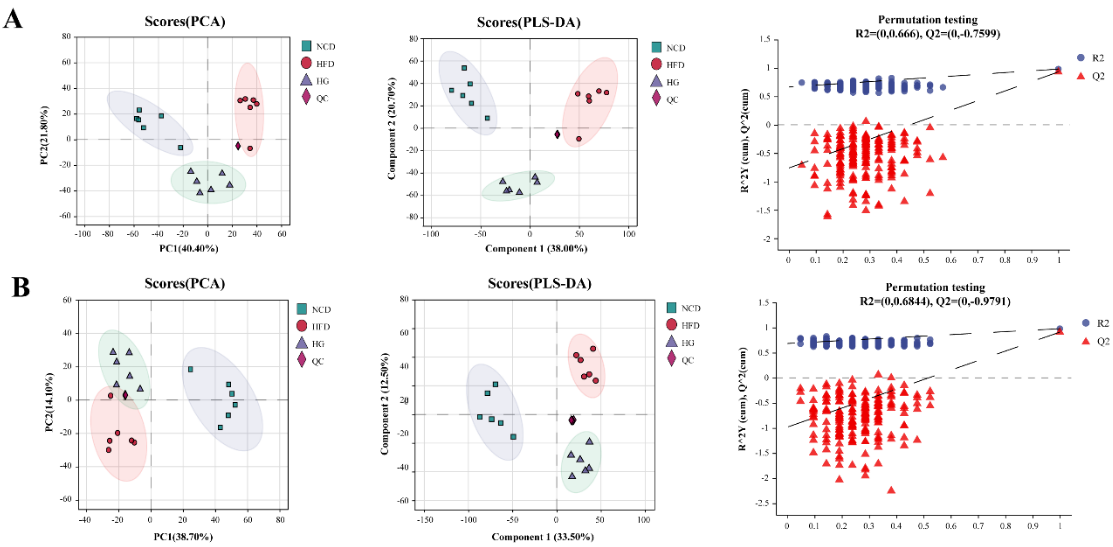

Figure S3. Fecal non-targeted metabolomics analysis of GL-treated *ApoE*$^{-/-}$ mice. (**A**) The PCA and PLS-DA score plots for each group (feces) in positive ion mode and corresponding permutation test. (**B**) The PCA and PLS-DA score plots for each group (feces) in negative ion mode and corresponding permutation test.

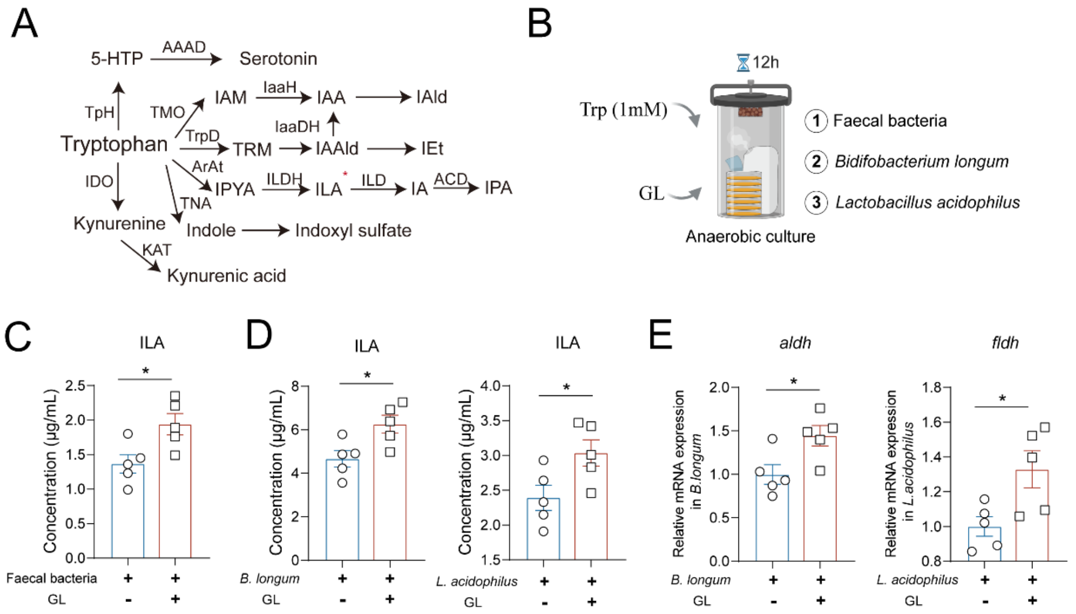

Figure S4. GL promotes ILA production by gut microbiota. (**A**) The pathways of microbial tryptophan metabolism. (**B**) Schematic of the experimental design for *in vitro* anaerobic incubation of fecal microbiota and representative ILA-producing strains (*B. longum* and *L. acidophilus*). (**C**) ILA concentrations in the culture supernatants of fecal microbiota following a 12 h incubation with or without GL. (**D**) ILA concentrations in the culture supernatants of *B. longum* and *L. acidophilus* mono-cultures treated with or without GL for 12 h. (**E**) Relative mRNA expression levels of *aldh* in *B. longum* and *fldh* in *L. acidophilus* measured by RT-qPCR. n = 5. Data are presented as means ± SEM. Statistical significance was assessed using one-way ANOVA. * $p < 0.05$. Abbreviations: 5-HTP, 5-hydroxytryptophan; IAM, indole-3-acetamide; IAA, indole-3-acetic acid; IAld, indole-3-carboxaldehyde; TRM, tryptamine; IAAld, indole-3-acetaldehyde; IEt, tryptophol; IPYA, indole-3-pyruvate; ILA, indole-3-lactic acid; IA, indole-3-acrylic acid; IPA, indole-3-propionic acid; TpH, tryptophan hydroxylase; AAAD, aromatic L-amino acid decarboxylase; IDO, indoleamine 2,3-dioxygenase; KAT, kynurenine aminotransferase; TMO, tryptophan 2-monooxygenase; IaaH, indoleacetamide hydrolase; TrpD, tryptophan decarboxylase; IaaDH, indoleacetaldehyde dehydrogenase; ArAT, aromatic amino acid aminotransferase;

ILDH, indolelactate dehydrogenase; ILD, indolelactate dehydratase; ACD: acyl-CoA dehydrogenase; TNA, tryptophanase.

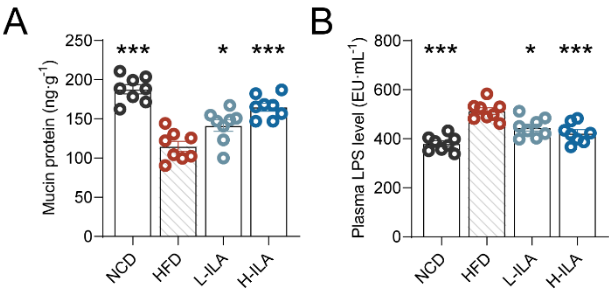

Figure S5. ILA restored colonic mucin protein and reduced LPS levels. (**A**) The level of mucin protein (n = 8). (**B**) The level of LPS in plasma (n = 8). Data are presented as means ± SEM, * $p < 0.05$, *** $p < 0.001$, using ANOVA.

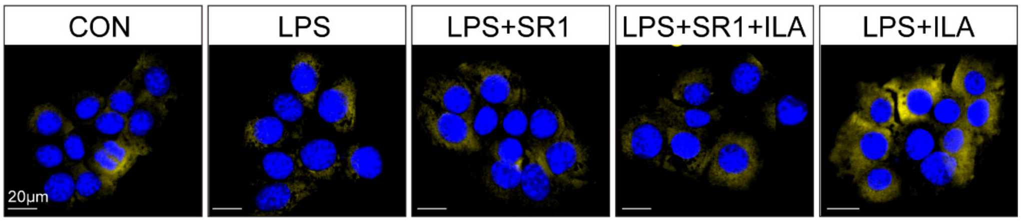

Figure S6. Immunofluorescence analysis of AhR in HAECs (scale bar, 20 μm).

Table S1. Reagents.

Reagent	Sources	Identifier
**Antibodies**		
Anti-VCAM-1	Abcam	ab134047
Anti-ICAM-1	Proteintech	10020-1-AP
Anti-ZO-1	Proteintech	21773–1-AP
Anti-OCLN	Proteintech	27260–1-AP
Anti-AhR	Proteintech	28727-1-AP
Anti-p65	Proteintech	66535-1-Ig
Anti-p-p65	CST	3031S
Anti-GAPDH	Beyotime	AF1186
HRP-conjugated Anti-Rabbit IgG(H+L)	Beyotime	A0208
HRP-conjugated Anti-Mouse IgG(H+L)	Beyotime	A0216
Alexa fluor® 594 conjugated donkey anti-rabbit IgG	Thermofisher	R-37119
IF488-Tyramide amplification	Servicebio	G1231
CY3-Tyramide amplification	Servicebio	G1223
CY3-conjugated goat anti-rabbit secondary antibody	Thermofisher	A10520
FITC-conjugated goat anti-mouse secondary antibody	Thermofisher	A16079
**ELISA kit**		
Mouse ICAM-1 ELISA Kit	Elabscience	E-EL-M3037
Mouse VCAM-1 ELISA Kit	Elabscience	E-EL-M1233
Mouse MCP-1 ELISA Kit	Elabscience	E-EL-M3001
Mouse ox-LDL ELISA Kit	Elabscience	E-EL-M0066
Mouse Aldosterone ELISA Kit	Elabscience	E-EL-0070
Mouse Renine ELISA Kit	RayBiotech	P06281
**Other Reagents**		
vancomycin	Sangon Biotech	A414413
neomycin sulfate	Sangon Biotech	A610366
metronidazole	Sangon Biotech	A600633
amphotericin B	Sangon Biotech	A429759
Stemregenin 1	MCE	HY-15001
Glycyrrhizic acid	MCE	HY-N0184
Lipopolysaccharides	MCE	HY-D1056
4-Nitrophenyl a-D-glucopyranoside	MCE	HY-W011411
Indole-3- lactic acid	MCE	HY-113099
Indole-3-acetic acid	MCE	HY-18569
Tryptophol	MCE	HY-W010155
Indole-3-propionic acid	MCE	HY-W015229
Indole-3-acrylic acid	MCE	HY-W015273
Indole-3-carboxaldehyde	MCE	HY-W007376
endothelial cell medium	ScienCell	1001
penicillin-streptomycin (P/S) solution	ScienCell	0503
fetal bovine serum	ScienCell	0025
endothelial cell growth supplement	ScienCell	1052
0.25% trypsin-EDTA	Gibco	C25200056
RPMI 1640	Gibco	C22400500BT
DPBS	Gibco	C14190500BT
PBS	Gibco	C10010500BT

Table S2. The primer sequences used in RT-qPCR.

	Gene	Forward Primer	Reverse Primer
Mouse	*Gapdh*	CTCCCACTCTTCCACCTTCG	TAGGGCCTCTCTTGCTCAGT
	Icam1	AAACCAGACCCTGGAACTGCAC	GCCTGGCATTTCAGAGTCTGCT
	Vcam1	CACTTGTGGAAATGTGCCCG	TCACACTCGTATATGCCGGC
	Mcp1	GCTACAAGAGGATCACCAGCAG	GTCTGGACCCATTCCTTCTTGG
	TNFα	CACAGAAAGCATGATCCGCG	ACTGATGAGAGGGAGGCCAT
	IL6	AGCCAGAGTCCTTCAGAGAGA	GGATGGTCTTGGTCCTTAGCC
	IL1β	CAGCTGGAGAGTGTGGATCC	TGCTTGTGAGGTGCTGATGT
	Muc2	TTCAAGAAGCTAACATGTGTGC	GGTAACTTGTGTTTCACAGTCC
	Muc4	AAGCGTGTTGCCTCTTCACCTG	AGGTGGCTGAGACTGCTGTGTT
	Zo1	AAACAGCCCTACCAACCTCG	CCTCTGGCCTGTACCTGTTG
	Ocln	TCTCTCAGCCAGCGTACTCT	TCCCATAGCCTCTGTCCCAA
	Cldn1	GGACTGTGGATGTCCTGCGTTT	GCCAATTACCATCAAGGCTCGG
	F11r	CACCTACTCTGGCTTCTCCTCT	TGCCACTGGATGAGAAGGTGAC
Human	*GAPDH*	GGTGAAGGTCGGAGTCAACG	TGGGTGGAATCATATTGGAACA
	ICAM1	AGCGGCTGACGTGTGCAGTAAT	TCTGAGACCTCTGGCTTCGTCA
	VCAM1	GATTCTGTGCCCACAGTAAGGC	TGGTCACAGAGCCACCTTCTTG
	TNF	CTCTTCTGCCTGCTGCACTTTG	ATGGGCTACAGGCTTGTCACTC
	IL6	AGACAGCCACTCACCTCTTCAG	TTCTGCCAGTGCCTCTTTGCTG
	IL1B	CCACAGACCTTCCAGGAGAATG	GTGCAGTTCAGTGATCGTACAGG
B. longum	*adlh*	TCTCAAGACCATCATCGGCG	GCGAAACGGTCTTGGTTGTC
L. aci-dophilus	*fldh*	ACCAGACGTTCCAGCTAACG	GCCGAAGATCTTGCCTGAGT
Bacteria	*16s rRNA*	ACTCCTACGGGAGGCAGCAG	ATTACCGCGGCTGCTGG
